# P-1408. Prevalence and risk factors of latent tuberculosis infection among patients with diabetes mellitus in a tertiary care teaching hospital in Nepal

**DOI:** 10.1093/ofid/ofaf695.1595

**Published:** 2026-01-11

**Authors:** Sailesh Shrestha

**Affiliations:** NYC Health + Hospitals/Harlem, New York, NY

## Abstract

**Background:**

Diabetes mellitus (DM) increases the risk of latent tuberculosis infection (LTBI) and its progression to active disease, leading to poorer treatment outcomes and higher relapse rates. Identifying and treating LTBI in people with DM offers a critical opportunity to reduce the TB burden. In Nepal, where both TB and DM are prevalent, data on LTBI in this group are limited. This study aims to estimate the prevalence and risk factors of LTBI among diabetic patients in a tertiary care hospital in Nepal.

**Methods:**

We conducted a cross-sectional study at the Department of Internal Medicine, Bir Hospital, National Academy of Medical Sciences, Kathmandu, Nepal. We enrolled 120 adult patients, determined a priori, with DM attending the outpatient and inpatient units, excluding those with a history of active TB, immunocompromising conditions, or pregnancy. We collected demographic and clinical data through interviews, physical examinations, and medical record reviews. We screened all participants for TB symptoms; those with symptoms underwent chest X-ray and GeneXpert testing to rule out active TB (Figure 1). We administered the Mantoux tuberculin skin test (TST) to participants without symptoms or with negative TB evaluations. We defined LTBI as an induration of ≥10 mm in the absence of active TB. We analyzed the data using R, reported LTBI prevalence as a proportion, and used logistic regression to identify associated risk factors.

**Results:**

Of the 157 eligible patients identified, 128 patients were included in the analysis (Figure 2). The median age of the study participants was 55.78 (IQR 46 – 65) years, and male and female populations were nearly equal (Table 1). 52 patients had TST induration of 10 mm or more, giving the LTBI prevalence of 40%. In the multiple logistic regression analysis, higher HbA1C levels, insulin use, and longer duration of diabetes were independently associated with increased odds of latent tuberculosis infection among patients with diabetes (Table 2).

**Conclusion:**

LTBI was common among patients with DM in the Nepalese tertiary care setting and was significantly associated with poor glycemic control, insulin use, and longer DM duration, suggesting that these patient populations could be a potential target for LTBI screening and prevention strategies.

**Disclosures:**

All Authors: No reported disclosures

